# Plasmonic Properties of Self-Assembled Gold Nanocrescents: Implications for Chemical Sensing

**DOI:** 10.1021/acsanm.4c00258

**Published:** 2024-04-08

**Authors:** Marie-Pier Côté, Christina Boukouvala, Josée Richard-Daniel, Emilie Ringe, Denis Boudreau, Anna M. Ritcey

**Affiliations:** † Department of Chemistry, Center for Optics, Photonics and Lasers, and Center for Research on Advanced Materials, Laval University, Quebec City G1 V 0A6, Canada; ‡ Department of Materials Science and Metallurgy and Department of Earth Sciences, University of Cambridge, Cambridge CB3 0FS, United Kingdom

**Keywords:** metal nanoparticles, gold nanostructures, block copolymer, self-assembly, nanocrescents, localized surface plasmon resonance

## Abstract

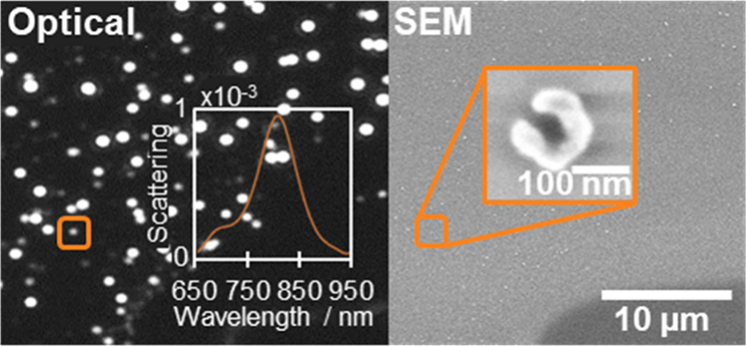

A bottom-up approach, the Langmuir–Blodgett technique, is used for the preparation of composite thin films of gold nanoparticles and polymers: poly(styrene-*b*-2-vinylpyridine), poly-2-vinylpyridine, and polystyrene. The self-assembly of poly(styrene-*b*-2-vinylpyridine) at the air–water interface leads to the formation of surface micelles, which serve as a template for the organization of gold nanoparticles into ring assemblies. By using poly-2-vinylpyridine in conjunction with low surface pressure, the distance between nanostructures can be increased, allowing for optical characterization of single nanostructures. Once deposited on a solid substrate, the preorganized gold nanoparticles are subjected to further growth by the reduction of additional gold, leading to a variety of nanostructures which can be divided into two categories: nanocrescents and circular arrays of nanoparticles. The optical properties of individual structures are investigated by optical dark-field spectroscopy and numerical calculations. The plasmonic behavior of the nanostructures is elucidated through the correlation of optical properties with structural features and the identification of dominant plasmon modes. Being based on a self-assembly approach, the reported method allows for the formation of interesting plasmonic materials under ambient conditions, at a relatively large scale, and at low cost. These attributes, in addition to the resonances located in the near-infrared region of the spectrum, make nanocrescents candidates for biological and chemical sensing.

## Introduction

Gold nanoparticles (AuNPs) are of interest to a range of scientists because of their unique physicochemical and optical properties.^1^ For example, AuNPs offer facile surface functionalization, biocompatibility, and modulable plasmonic properties through the so-called localized surface plasmon resonances (LSPRs) that can be modified by varying particle shape^2^ and size.^3^ Together, these properties make AuNPs an attractive basis for the development of new sensing platforms. Furthermore, interparticle coupling, which arises when neighboring particles are brought into close proximity, leads to a greater sensitivity of the plasmon frequency to local environment than that of single, isolated particles.^4^ Therefore, creating nanostructures (NSs) composed of well-ordered NPs is beneficial for sensing applications.

**Figure 1:**
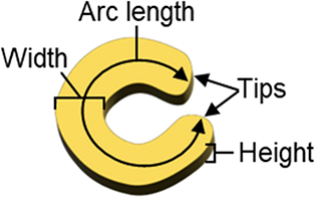
Representation of the dimensions of a nanocrescent.

Among existing NSs, nanocrescents have attracted considerable attention since they present LSPRs situated in the near-infrared (near-IR), leading to enhanced sensitivity to the surrounding medium.^5^ As illustrated in Figure 1, the nanocrescent structure consists of an incomplete ring, displaying a split or a gap that results in the formation of two extremities, known as the tips. The resonant plasmon frequencies of such structures depend on the arc length, the tip sharpness, and the width of the structure, i.e., the difference between the outer and inner diameter.^6^ For sizes below 25 nm,^7^ nanocrescents support pure dipolar plasmons. When the dimensions of the nanocrescent reach 60 nm and higher, the coupling effect becomes more complex since higher-order modes arise.^7^ Further complexity is added when Fano resonances appear in the extinction spectra. A Fano resonance occurs when a discrete (subradiant) and a continuous state (superradiant) overlap and interfere,^8^ leading to a spectral feature exhibiting a strong dependence on the local environment that can be harnessed in sensing applications.^9−11^ As reported elsewhere,^11^ Fano resonances in nanocrescents are influenced by the tip-to-tip distance as well as the height of the structure and result from interference between the dipolar mode of the tips along the height axis (acting as the discrete state) and the quadrupolar mode within the plane of the crescent (which constitutes the continuous state). The sensitivity of the plasmonic response of nanocrescents to their dimensions leads to highly tunable optical properties that provide versatility for many applications such as biological and chemical sensing, nanomedicine, nanolasing, and catalysis.^5,8,9,12−26^ For example, Park et al. fabricated nanocrescent antennas on mesoporous silica nanospheres for cellular imaging, molecular targeting, and drug delivery.^25^ Zhang and co-workers demonstrate the potential of gold nanocrescents in asymmetric catalysis and as a surface-enhanced Raman scattering (SERS) platform for the chiral detection of molecules.^26^

To the best of our knowledge, gold nanocrescents have so far only been obtained by using a combination of bottom-up and top-down approaches^5,12,14−23,25−27^ or top-down approaches alone.^8,13,24,28−30^ Several of these studies include the correlation of plasmonic properties with structural features such as the size, aspect ratio, tip sharpness, and arc length of the nanocrescents.^15,18,24,30^ Despite these advances, the development of an inexpensive sensing platform composed of large-scale, well-ordered metallic NSs remains challenging.^21,30,31^ In addition, it is also desirable to decrease the number of experimental steps required to prepare the structures.^12^ Bottom-up approaches based on self-assembly are particularly attractive, despite their tendency to present a greater heterogeneity than top-down approaches.^32^ The development of a completely bottom-up method for the preparation of plasmonic NSs would therefore be advantageous since it would allow for scalable production at low cost and high throughput.

Block copolymers (BCPs) can form highly ordered, periodic structures extending over large length scales through the spontaneous microphase separation of immiscible blocks and have thus emerged as a key self-assembly tool.^33^ BCPs such as poly(styrene-*b*-poly(methyl methacrylate)) (PS-*b*-PMMA),^34,35^ poly(styrene-*b*-4-vinylpyridine) (PS-*b*-P4VP), and polystyrene-*b*-poly-2-vinylpyridine (PS-*b*-P2VP)^36^ form surface micelles when spread at an air–water interface and have been used as templates for the organization of small (5–7 nm) AuNPs with the Langmuir–Blodgett (LB) technique. Among these polymers, PS-*b*-P2VP, when spread at the air–water interface, forms a hexagonal arrangement of surface micelles with the greatest order.^36^

Here, the bottom-up LB self-assembly method is used to prepare composite ultrathin films containing small AuNPs organized into nanorings within a BCP matrix.^35,36^ Subsequent growth^37^ of the AuNPs through the reduction of additional gold leads to circular arrays of larger AuNPs (CAs) and nanocrescents. Furthermore, by tuning the self-assembly parameters, the distance between the NSs can be increased beyond the diffraction limit and allow for the optical characterization of individual structures by dark-field microscopy. Using marked substrates, spectral signatures recorded from single entities can be attributed to specific NSs imaged with scanning electron microscopy (SEM), providing unprecedented structure–property correlations. The experimental results are compared with electromagnetic simulations that provide additional insight into the plasmonic response as a function of NS morphology, as well as visualization of near-field effects. Of the various NSs investigated, the nanocrescents exhibit the most exciting plasmon properties relevant to the development of a sensitive and inexpensive sensing platform.

## Experimental Section

### Materials

Poly(styrene-*b*-2-vinylpyridine) (*M*_n_: (55,000-*b*-50,000) g/mol, PI: 1.05), poly-2-vinylpyridine (*M*_n_: 3,700g/mol, PI: 1.44), and polystyrene (*M*_n_: 52,000 g/mol, PI: 1.07) were purchased from Polymer Source TM Inc. Methanol (99.9%) and hydrogen peroxide (30%) were obtained from Fischer Scientific. Ammonium hydroxide (28%) was provided by Caledon. All other chemicals were acquired from Sigma-Aldrich: tetrachloroauric(III) acid trihydrate (≥99.9% trace metals basis), cetyltrimethylammonium bromide (≥98%), ascorbic acid (99%), 1-octanethiol (98%), chloroform (≥99%, HPLC grade), sodium borohydride (Sigma-Aldrich, 99%), tetraoctylammonium bromide (98%), and Triton X-100 (nonionic, laboratory grade). A Nanopure II filtration system was used to obtain ultrapure water. Transmission electron microscopy (TEM) finder grids Maxtaform Style H2 (nickel) were purchased from Ted Pella Inc.

### Synthesis of AuNPs

AuNPs synthesis is based on a modified Brust–Schiffrin method.^38^ The first step involves mixing 100 mL of the organic phase containing a phase transfer agent (64.0 mM tetraoctylammonium bromide in chloroform) with 50 mL of the aqueous phase (30.5 mM gold(III) chloride trihydrate in ultrapure water). The mixture was stirred until the aqueous phase became colorless and the organic phase became red, indicating the complete transfer of tetrachloroaurate ions from the aqueous solution to the organic phase. Then, 20 mL of sodium borohydride (0.833 M) dissolved in ultrapure water was added in one shot to reduce the gold salt. The organic solution was separated from the aqueous solution by decantation and washed once with 0.1 M sulfuric acid in ultrapure water and twice with ultrapure water. Octanethiol was added in excess (1 mL) to the organic solution. The functionalization of AuNPs was achieved under vigorous stirring maintained overnight. The AuNPs were purified by three centrifugations (15,000 rpm/22640 RCF, 30 min) in chloroform/methanol to remove excess ligands. Finally, the NPs were dried, weighed, and stored in a dry, cool, and dark place.

### Substrate Preparation

Glass coverslip substrates were cleaned before use with the following procedure. The substrates were first sonicated in a Triton X-100 solution (10% in ultrapure water) for 30 min. They were then thoroughly rinsed with ultrapure water before being placed in a base-piranha solution (1 H_2_O_2_:1 NH_4_OH:5 H_2_O) for 2 min at 90 °C followed by final rinsing with ultrapure water. In order to facilitate the identification of individual NSs with SEM and optical scattering, the glass substrates were patterned with a carbon film. To achieve this, TEM finder grids, with a grid bar width of 19 μm and spacings of the order of 100 μm, serving as a mask, were placed on a portion of the glass coverslip before the addition of a carbon coating by a physical vapor deposition technique. The TEM finder grids were removed after carbon deposition to expose the underlying glass and obtain the marked substrates employed for SEM and dark-field microscopy measurements. In addition, carbon-coated TEM grids were glued onto the glass coverslips before the monolayer deposition to permit the TEM observation of a portion of the sample.

### Preparation of LB Films

The self-assembled NSs were prepared with a KSV NIMA 3000 LB instrument using a slightly modified version of a previously reported method.^35^ Basically, a solution composed of approximately 30.0 mg of P2VP and 18.0 mg of PS-*b*-P2VP in 10 mL of chloroform was prepared. Then, 1 mL of this solution was added to 2.5 mg of AuNPs and mixed in an ultrasonic bath for 1 min. The spreading solutions were used within 1 day of their preparation. Using a microsyringe, approximately 4 μL of the resulting solution was spread dropwise on the surface of an ultrapure water subphase in the Langmuir trough. The monolayers were compressed to a surface pressure of 1 mN/m at a rate of 5 mm/min at room temperature. The compression isotherm of the composite film is provided in Figure S2. After a delay of 30 min from the time of spreading, the compressed films were transferred on the upstroke at a speed of 5 mm/min to solid substrates immersed in the subphase prior to monolayer spreading. The transferred LB films were dried under an air flow and kept covered to protect from dust.

### In Situ Regrowth Method

The growth procedure described here has been adapted from a previously reported method.^37^ Cetyltrimethylammonium bromide (93.8 mM) and tetrachloroauric(III) acid trihydrate (60.9 μM) were dissolved in ultrapure water at 30 °C. A 10 mL aliquot of this solution, which is light orange in color, was transferred into a 30 mL polypropylene vial and cooled in an ice–water bath. After the solution had reached a temperature of 15 °C, 50 μL of 0.1 M l-ascorbic acid in ultrapure water was added under stirring. Stirring was stopped once the initial orange color vanished (approximately 1 min), and the substrate-supported NSs were immersed for 10 min. Lastly, the samples were rinsed with ultrapure water, submerged in ultrapure water for 15 min, and dried under flowing air.

### Characterization

TEM images of the NSs after AuNPs growth were obtained with an FEI Tecnai G2 Spirit Biotwin transmission electron microscope. SEM images were obtained with an FEI QUANTA-3D-FEG. NS dimensions, and interstructure distances were determined from SEM images with ImageJ software. Scattering spectra were recorded from individual NSs with the hyperspectral dark-field microscopy technique using an inverted optical microscope coupled with a spectrometer as described in detail elsewhere.^39^ Spectra obtained were smoothed by using the moving average method.

### Numerical Calculations

Optical scattering spectra were obtained numerically in the discrete dipole approximation using DDSCAT.^40,41^ AutoCAD 2020 was used to create a 3D model of each NS, based on SEM images, which was then converted into the required dipole array for DDSCAT using a script developed in MATLAB. Due to this approach, the modeled NSs have sufficiently accurate lateral dimensions; however, their height had to be estimated. The frequency-dependent refractive index of metallic Au was taken from Johnson and Christy^42^ and the ambient refractive index (RI) was set to 1.59 for polystyrene.^43^ Unless stated otherwise, for each simulation, two incident orthogonally polarized field directions forming an angle of 31° to the substrate and perpendicular to each other were used to approximate the unpolarized light and the light cone generated by the condenser of the dark-field microscope setup. Scattering efficiency, obtained from DDSCAT, is defined as *C*_sca_/πα_eff_^2^, where *C*_sca_ is the scattering cross section and α_eff_ the radius of a sphere of the same volume as the nanostructure. All calculations were carried out with dipole distances from 1 to 2 nm. Electric field distributions were plotted at the phase with the highest field intensity. Further numerical calculation parameters can be found in Tables S1 and S3.

## Results and Discussion

### Nanostructure Assembly and Secondary Growth

**Figure 2:**
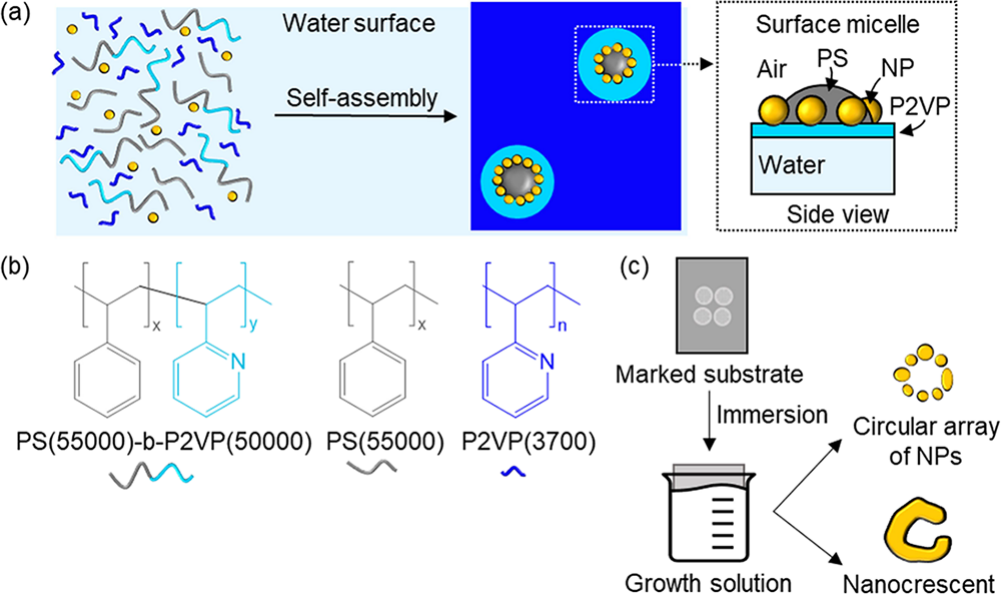
Schematic illustrating the (a) self-assembly of AuNPs and block copolymers at the air-water interface, (b) chemical structure of the polymers used with their respective number-average molecular weight and color-coded gray (PS and h-PS), light blue (P2VP), and blue (h-P2VP), and (c) NSs obtained after the subsequent growth of the transferred NSs.

As sketched in Figure 2, amphiphilic block copolymers can form surface micelles at the air–water interface.^35^ As described elsewhere, small (∼4–6 nm) AuNPs coated with octanethiol, when cospread with PS-*b*-P2VP, self-organize into rings around the hydrophobic polystyrene (PS) domains of the surface micelles.^35,36^ In order to capture the light scattered from individual NSs by dark-field microscopy, it is imperative that they be separated by distances greater than the diffraction limit. For this reason, the distance between the micelles was increased by the addition of the P2VP homopolymer (h-P2VP) and the application of a low surface pressure (1 mN/m) during film transfer to the glass substrate. Unexpectedly, adding h-P2VP also leads to a decrease in the size of the BCP surface micelles from 45 to 25 nm for h-P2VP concentrations ranging from 0 to 7 mg/mL (data presented in Figure S1). To counteract this effect, a small amount of polystyrene homopolymer (h-PS) was also added to obtain surface micelles comparable to the close-packed ones. The addition of h-PS swells the PS domains and leads to larger sizes, which is consistent with the work of Wen et al.^44^

**Figure 3:**
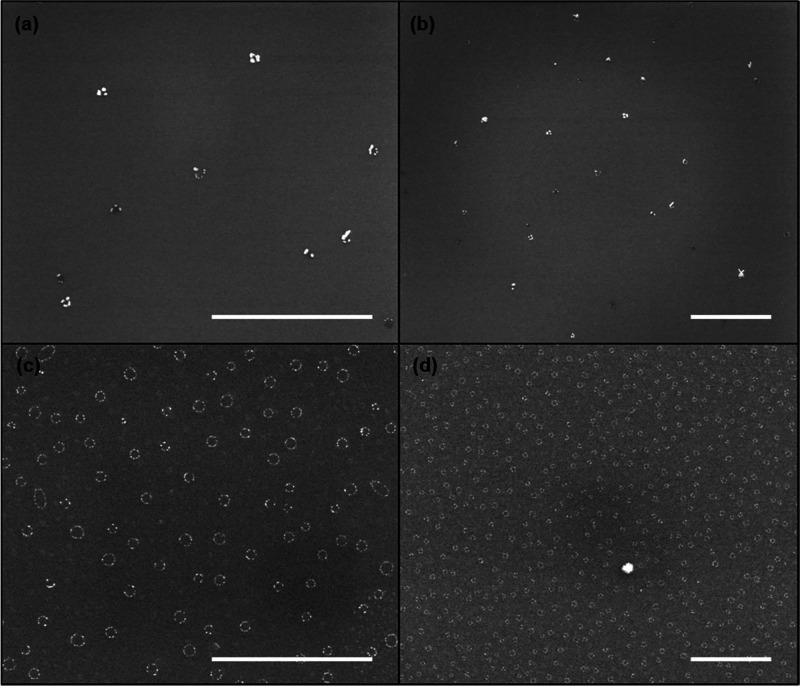
SEM images at two different magnifications of NSs (a, b) on glass and (c, d) on carbon in different regions of the same sample. Scale bars are 2 μm.

The self-assembled composite films are transferred from the water surface to patterned glass/carbon substrates to enable the identification of specific individual NSs in both optical microscopy and SEM images. In the final step of sample preparation, the as-deposited NSs are subjected to a secondary growth process (illustrated in Figure 2c) to increase particle size and decrease the interparticle distance, leading to a higher intensity of plasmonic scattering as well as the appearance of new coupled modes. SEM images of the deposited NSs after secondary growth on a hybrid patterned glass/carbon substrate are presented in Figure 3. Although NSs are present across the entire sample, the carbon-coated regions surprisingly display much denser arrays than those on glass. Moreover, when the amount of h-P2VP used for the self-assembly is above 1 mg/mL, the separation between CAs becomes irregular and areas without CAs can be found, potentially occupied by h-P2VP only. Further details concerning the influence of various parameters on NS spacing are provided in Section S2 of the Supporting Information. The irregular spacing of the CAs is potentially due to the dominance of the h-P2VP–glass interactions through hydrogen bonding^45^ and the rupture of the film during the dewetting process (Figure S5). This results in a higher probability of finding sufficiently spaced NSs on glass than on carbon. NSs deposited on glass are therefore ideal for optical far-field characterization since they reach spacings between 350 nm and 2.2 μm, which is larger than the diffraction limit of the optical microscope, allowing for the resolution of individual structures.

**Figure 4:**
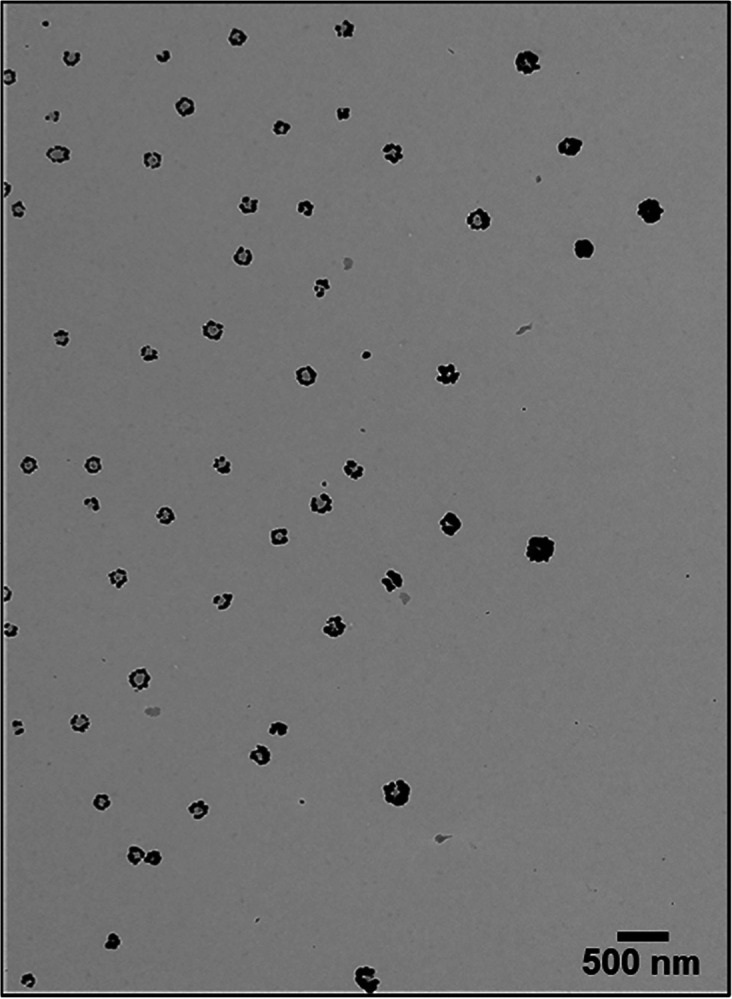
TEM images of NSs after regrowth on a TEM grid that was fixed to a glass substrate during film transfer from the air–water interface.

As illustrated in Figure 4, in situ regrowth leads to a gradient in NS size, with the final size of each structure being inversely related to the proximity of neighboring structures. It is important to note that the irregular growth of the NSs is a direct result of the irregular interstructure spacing rather than the nature of the substrate. Figure 3c,d shows that more uniform structures are obtained when the distance between them is relatively constant. Irregular regrowth is thus an undesirable consequence of the necessity to separate the NSs sufficiently for their characterization with dark-field microscopy. Structures prepared for eventual applications, on the other hand, would be close-packed and, therefore, more regular. The size variation observed in Figure 4 can be attributed to the increase in the number of precursor ions available for the growth of more highly separated NSs due to the diffusion-limited nature of the process. Indeed, the high concentration of CTAB (80 mM) defines the system as diffusion-limited rather than as reaction-limited as found at lower CTAB concentrations.^46^

Although undesirable for the homogeneous coverage of large surface areas with uniform structures, the observed gradient in NS growth provides a plethora of structures within a single sample, which is ideal for the fundamental investigation of shape effects on the plasmonic properties. Within the obtained NSs, we distinguish between two morphological categories: NPs that have merged into crescent-like shaped NSs (nanocrescents),^48^ also described as split nanorings in the literature,^47^ and NSs that display three or more well-distanced NPs forming a circular array of NPs (CAs).

### Optical Properties and Structure Correlation of Nanocrescents

Scattering spectra of individual nanocrescents were collected by dark-field scattering microscopy coupled to a spectrometer for hyperspectral imaging using a 100X oil immersion objective, as described previously.^39^ The use of a marked substrate allowed for identification and SEM imaging of the specific structure responsible for each individual scattering spectrum. Around 125 NSs were analyzed, with selected representative examples presented here. Lower-magnification SEM images of each of the NS presented in the main paper are reported in Figures S6 and S7.

**Figure 5:**
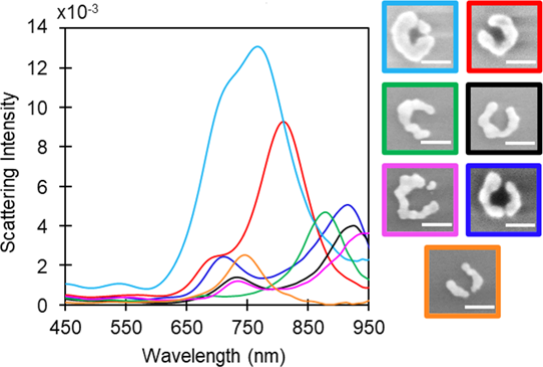
Representative SEM images of isolated nanocrescent structures along with their corresponding light blue, red, green, black, pink, blue, and orange color-coded dark-field scattering spectra. Scale bars are 50 nm.

**Figure 6:**
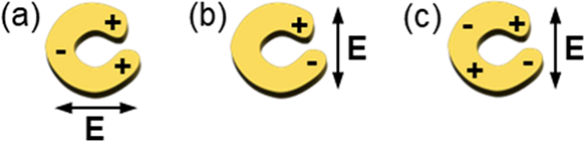
Polarization-dependent excitation of plasmon modes in nanocrescents, (a, b) dipolar in both polarizations and (c) quadrupolar when the polarization is parallel to the gap, modified from Clark et al.^30^

The results presented in Figure 5 illustrate how details of the nanocrescent morphology, such as size and asymmetry, significantly influence the plasmonic interaction with light, resulting in distinct spectral signatures covering the visible and near-IR ranges. For most of the structures, two distinct peaks are observed in the experimental spectrum. Similar plasmonic spectra have been reported for other nanocrescent structures.^15,18,24,30^ Of these, it is the gold crescent-shaped split-ring resonators with arc lengths between 450 and 675 nm, fabricated by electron beam lithography and reported by Clark et al.,^30^ that most resemble in shape the NSs shown in Figure 5. Through modeling, these authors were able to assign the low-and high-energy peaks to first- and third-order modes, respectively, for light polarized parallel to the gap. Furthermore, the higher-order resonance was found to vanish for nanocrescents with arc lengths below 500 nm. In the case of polarization perpendicular to the gap, a single second-order mode was observed, situated at a frequency similar to that predicted for the third-order resonance observed for parallel polarization.^30^ These polarization-dependent plasmon modes are illustrated in Figure 6.

Based on the assignments of Clark et al.,^30^ the observed high- and low-energy resonances in the experimental spectra of Figure 5 can tentatively be assigned to dipolar modes perpendicular and parallel to the gap, respectively. Both modes can be excited because the optical response is probed with unpolarized light. The experimental spectra of Figure 5 are also significantly blue-shifted with respect to those reported by Clark et al., as would be expected given the smaller dimensions of the nanocrescents investigated here (Table S1). Furthermore, since the dark-field microscopy measurements are performed with an angle of incidence of 31°, possible out-of-plane contributions must also be considered. This point is addressed in the calculations described below.

**Figure 7:**
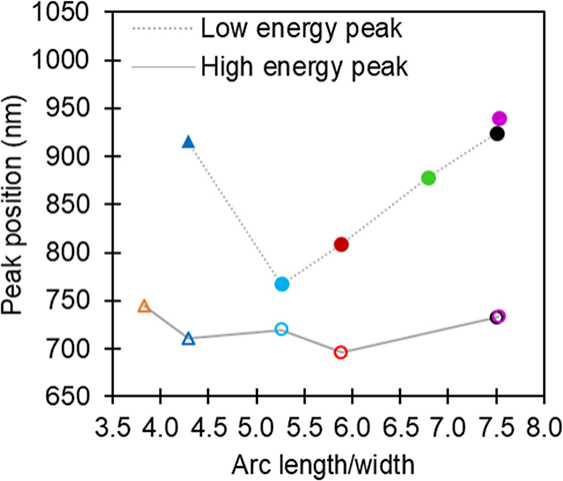
Position of scattering maxima (λ_max_) as a function of arc length-to-width ratio (values are provided in Table S2). Colors correspond to the color-coded nanostructures in Figure 5. Circular and triangular symbols represent nanocrescent and split-ring NSs, respectively. Filled and open symbols correspond to the position of low- and high-energy peaks, respectively. Lines have been added only to guide the eye.

Analysis of the extinction spectra and NSs of Figure 5 reveals that the position of the high- and low-energy peaks depends primarily on the width and the arc length of the nanocrescents, which is consistent with the literature.^30,49^Figure 7 highlights the wavelength dependence with increasing arc length (*l*) and decreasing width (*w*). Therefore, we considered the *l*/*w* ratio and found that the LSPR response red-shifts as the *l*/*w* ratio increases. Specifically, the low-energy peak redshifts in the order of the light blue (∼767 nm), red (∼809 nm), green (∼878 nm), black (∼925 nm), and pink (∼940 nm) spectra (color-coding of Figure 5), which correspond, respectively, to NSs with increasing *l*/*w* ratios of roughly 5.26, 5.89, 6.79, 7.50, and 7.53. The second lowest energy peak of nanocrescents red-shifts in the order of red (∼696 nm), black (∼733 nm), and pink (∼734 nm) spectra, following the same trend. The LSP frequency of the light blue nanocrescent (∼720 nm, *l*/*w* = 5.26) does not follow this trend as it is higher than expected compared to the red NSs (∼696 nm, *l*/*w* = 5.89). This observation may be related to the presence of a discrete NP between the tips of the light blue NS, which modifies the plasmon coupling. The discrete NP may also be responsible for the additional resonance observed at 550 nm for this NS. The hypothesis that the high-energy resonance of the light blue NS is modified by the presence of an NP in the gap is, however, in contradiction with the assignment of this resonance to a dipolar mode perpendicular to the gap. The presence of an NP in the gap would rather be expected to perturb the low energy mode since it is attributed to excitation across the gap. The data presented in Figure 7 indicate, however, that the low-energy peak for the light blue structure perfectly follows the trend of the other nanocrescent structures. This point is revisited in the simulation section below.

Further analysis of the spectra reveals that the energy separation between the two dipolar modes (parallel and perpendicular to the gap) decreases as the crescent converges to a closed ring in the order of pink (Δλ ∼ 206 nm), black (Δλ ∼ 192 nm), red (Δλ ∼ 113 nm), and light blue (Δλ ∼ 47 nm) with tip-to-tip gaps of 35, 28, 24, and 12 nm, respectively. We hypothesize that the distance between the tips of the green nanocrescent (51 nm) is too large to sustain significant tip-to-tip coupling, which explains its singly peaked LSPR response. Meanwhile, the blue (*l*/*w* ratio of 4.29) and orange (*l*/*w* ratio of 3.83) structures behave differently presumably because of the two splits present in their structure and likely exhibit higher-order coupling modes; their LSPR responses thus do not follow the same trend as the other nanocrescents.

### Numerical Results for Various Crescent Heights

Numerical calculations of two NSs, referred to as the crescent and split-ring structures in red and blue boxes in Figure 5, were carried out for different height values in order to understand the plasmonic behavior of the nanocrescents. Calculated LSP energies correspond to contributions from in-plane polarization and out-of-plane polarization (Figure S8). The parameters used for the structures are summarized in Table S3.

**Figure 8:**
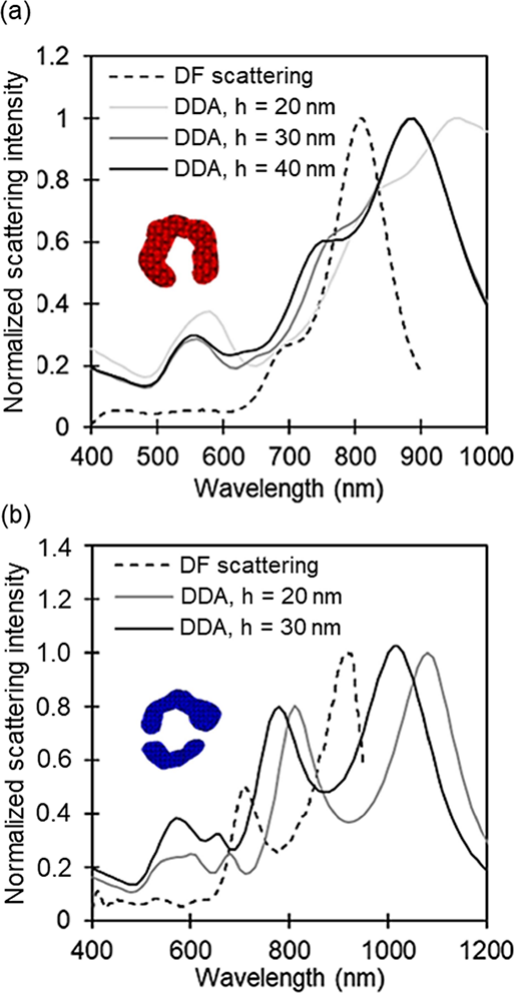
Normalized calculated scattering spectra of the crescent (a) and the split-ring (b) structures of different height values (solid lines) along with their dark-field scattering spectra (dashed line).

Overall, the numerical calculations predict a scattering profile similar to the experimental observations; however, there are also notable differences. Both experimental and calculated spectra exhibit two dominant peaks between 600 and 1200 nm. The higher intensity peak, experimentally found at about 810 nm for the crescent and 920 nm for the split ring, was calculated at 900 and 1060 nm for the 40 nm thick crescent and split-ring NS, respectively. In both cases, a similar shift of roughly 90 nm is observed between calculations and experiment, confirming the consistency of our modeling approach. The second higher energy peak appears as a shoulder for the crescent and a distinct peak for the split ring for both experiment and simulation and is again red-shifted for the latter. As demonstrated in Figure 8, height significantly influences the LSP peak position^9^ and can explain some of the discrepancies between the dark-field scattering and simulated spectra. Other parameters that could influence the plasmonic response are the unknown edge curvature,^50^ the influence of the substrate,^51^ and the tips of the nanocrescents that are rather rounded in contrast to the sharp ones commonly reported.^13,22−24,29,52^

### Field Distributions of the Nanocrescents

**Figure 9:**
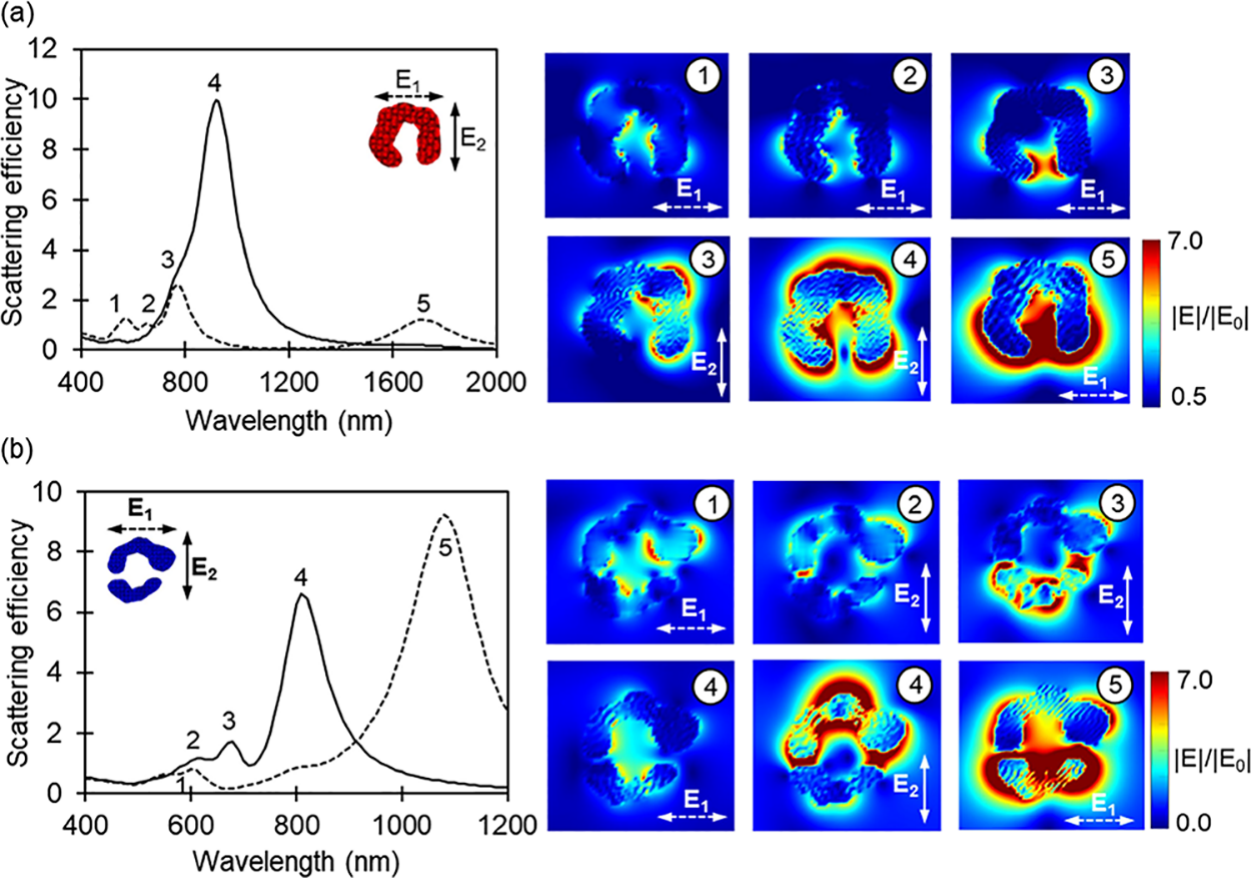
Calculated scattering spectra for two orthogonal polarizations (solid and dashed lines) for the 30 nm thick (a) crescent and (b) split-ring NSs along with field distributions corresponding to the most prominent LSPR peaks.

Electric field distribution maps were calculated for crescent and split-ring nanostructures in order to identify the plasmon modes responsible for the various resonances observed in the extinction spectra. The results are provided in Figure 9 along with the corresponding scattering spectra calculated for polarizations parallel and perpendicular to the structure gap. Additional phase-dependent charge distribution maps are provided in Figures S9 and S10.

When the nanocrescent is excited with light polarized parallel to the gap, four distinct peaks can be identified in the scattering spectrum (Figure 9a). At the highest energy (peak 1), the NS sustains a higher-order mode, but no tip-to-tip coupling is observed. Peak 2 corresponds to a second-order oscillation (quadrupole mode) coupled with an out-of-phase tip-to-tip dipole mode. The third peak coincides with a mode analogous to that sketched in Figure 6c. Although the charge distribution of this mode shares some attributes of a quadrupolar resonance, it cannot be identified as a true quadrupolar mode because of the absence of 4-fold symmetry in the field intensity. In the nanocrescent structure, the symmetry is broken by the gap. For this reason, it is perhaps more appropriate to describe the plasmon mode responsible for resonance 3 as a pair of coupled dipoles in which the electrons of the arc oscillate out-of-phase with the electrons of the tips. One important distinction between this mode and a true quadrupolar mode of a complete ring structure is the significant concentration of the electric field within the gap. The lowest energy band (peak 5) arises from a first-order oscillation (dipole mode) where electrons across the entire structure oscillate all in phase. There is also strong coupling between the tips.

For polarization perpendicular to the gap, the spectrum is dominated by the most intensely scattering mode sustained by the nanocrescent (peak 4), which corresponds to a dipole oscillating across the arc of the NS. A shoulder (peak 3) on the high energy side of the dipole peak emerges with strong field confinement on the right side of the structure. This feature is not observed for simulations carried out at normal incident (Figure S9), indicating that it is associated with the tilted propagating light direction.

Comparison of the simulated and experimental spectra of the nanocrescents leads to the assignment of the high- and low-energy resonances observed in Figure 5, respectively, to the peaks labeled 3 and 4 in Figure 9a. According to these assignments, it is the high-energy resonance (peak 3) that exhibits strong tip-to-tip coupling. It is therefore the high-energy resonance that is predicted to be perturbed by the presence of a discrete NP within the gap, consistent with what is observed for the light blue NS. The reassignment of the high-energy resonance to excitation parallel to the gap is also consistent with the observation that it is this resonance that vanishes when the gap becomes too large, as in the green nanocrescent in Figure 5. These results emphasize the importance of numerical simulations in the interpretation of the plasmonic properties of NSs.

The behavior of the split-ring NS gets more complex since additional coupling occurs considering the second tip present in the structure compared to the crescent with a single split. Peak 1 arises from a higher-order mode generated by out-of-phase oscillations. The right part of the top arc interacts strongly with light, behaving like a dipole in a nanosphere. For the second mode (peak 2), the upper arc behaves the same way as peak 1, but the intensity is higher. Similar to the crescent structure, strong tip-to-tip coupling is noted for the low-energy peak (peak 3). Interestingly, the electric field is stronger within the smaller part compared to the bigger one, presumably because the conduction electrons are confined inside smaller dimensions. In addition, the coupling is stronger between the tips on the left side of the NS. Strong interactions from the dipolar modes of the arc are observed and are stronger along the upper arc. That gives rise to a weak higher-order mode (peak 4). For light polarization perpendicular to the gap, the high-intensity/low-energy peak (peak 5) corresponds to the in-phase oscillations (dipole moment) of the NS. Again, the coupling is stronger within the smaller feature of the NS. As in Clark et al.,^30^ numerical results suggest that the two experimental plasmon bands (Figure 5) result from the combination of dipolar and quadrupolar modes along the tips and arc of the NS. Furthermore, the lowest energy peak (peak 5 in Figure 9), which substantially depends on the gap size, is outside of our experimental energy window.

### Correlation of the Optical Properties and Structure of the Circular Array of NPs

**Figure 10:**
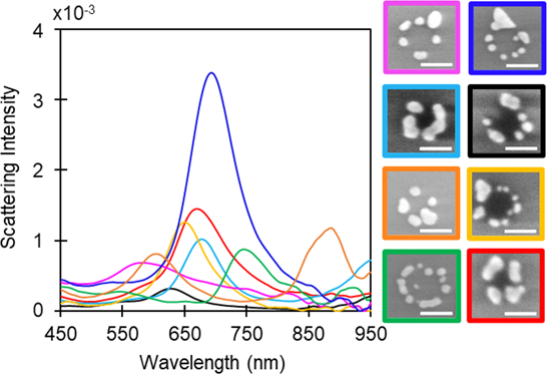
Representative SEM images of isolated CA NSs along with their corresponding pink, blue, light blue, black, orange, yellow, green, and red color-coded dark-field scattering spectra. Scale bars are 50 nm.

The correlation was conducted in the same way as that for the nanocrescents. The diversity of shapes and interparticle distance, as well as the position of particles within circular arrays (Figure 10), leads to a diversity of optical signatures. Their scattering intensity is 1 order of magnitude lower than that of the nanocrescents, as demonstrated both experimentally and numerically. They also interact mostly at lower wavelengths mainly by dipole coupling, as shown in Figure S11. Resonances appear at lower energies for NPs with a larger size and at higher energies for smaller NPs. Our measurements are in accordance with previous reports demonstrating that CA diameter, NP spacing, and NP dimensions greatly influence the LSPR response.^27,32,52^ Increasing the CA or NP diameter or decreasing the interparticle distance generally results in a redshift of the LSPR due to a stronger coupling between NPs. The morphology of the NPs also affects the spectral signature, as demonstrated in Figure 10 by the presence of triangle-like or rod-like NPs within the structures.

Numerical calculations show that the ease of matching the spectral signatures is reduced for nanoarrays compared to nanocrescents. This is likely due to the heterogeneity in NP size and shape forming the array, which we have shown to have a significant effect on the optical response (Figure S11). Nevertheless, numerical results can provide insight into the effects of the NP heterogeneity. For example, as shown in the simulations of Figure S12, a circular array of irregular particles exhibits numerous plasmon resonances, with lower scattering intensities than the corresponding arrangement of perfect disks, each with the same volume and at the same position as the irregular NPs of the NP array.

## Conclusions

We successfully prepared well-separated gold NSs with a copolymer template and the LB technique, which is a simple and low-cost method. NSs sufficiently distanced from each other to be resolved with optical scattering microscopy were obtained by the addition of a spacing agent (h-P2VP) and the low surface pressure applied at the air-water interface during transfer. Subsequent growth of the deposited assemblies leads to the formation of a variety of circular arrays of NPs and nanocrescents, allowing for the correlation of plasmon properties with the structure. Experimental results for representative examples show that the plasmonic response of nanocrescents exhibits two primary features: a more intense low-energy resonance and a less intense high-energy resonance. Numerical calculations led to the attribution of the low-energy resonance to a dipolar mode, oscillating in a direction perpendicular to the gap. The high-energy resonance is attributed to a quadrupolar-like mode that is excited by light polarized parallel to the gap and shows strong tip-to-tip coupling. Both resonances are red-shifted with an increasing arc length-to-width ratio. In addition, decreasing the gap width leads to a decrease in the separation of the two resonances.

Despite the inherent irregularities of the bottom-up structures, scattering spectra with well-defined resonances are obtained. This important result indicates that ring and crescent nanostructures do not have to be perfect to present potentially useful plasmonic properties. Nanocrescents are of particular interest since they scatter in the near-infrared and infrared region. In this case, it would be beneficial to develop a method for the fabrication of periodic arrays of gold nanocrescents using an entirely bottom-up approach since it allows for rapid fabrication at large scale and at a reasonable cost for sensing applications.

## Supporting Information Available

Influence of the addition of P2VP on the assemblies, details of the impact of surface treatment on the transfer of the NSs to solid substrates, SEM images of the isolated NSs and dimensions of the investigated NSs, parameters employed for numerical calculations, charge distribution maps calculated at normal incidence, electric field distribution of circular arrays of five NPs and nine NPs, and effect of morphological changes from perfect to actual paw structure on the LSPR response (PDF)
