# Supplementary material for: Plasmonic Properties of Self-Assembled Gold Nanocrescents: Implications for Chemical Sensing
Source: ACS Appl Nano Mater. 2024 Apr 8;7(8):8783–91. doi: 10.1021/acsanm.4c00258 (PMC11059077; doi:10.1021/acsanm.4c00258)
Supplement: Supplementary file 1 — an4c00258_si_001.pdf [file an4c00258_si_001.pdf]

Supporting Information for

Plasmonic Properties of Self-Assembled Gold Nanocrescents: Implications for Chemical Sensing

Marie-Pier Côté [α], Christina Boukouvala [β], Josée Richard-Daniel [α], Emilie Ringe [β], Denis Boudreau [α], Anna M. Ritcey [α]*

[α] Department of Chemistry, Center for Optics, Photonics and Lasers, and Center for Research on Advanced Materials, Laval University, Quebec City, Canada G1V 0A6.

[β] Department of Materials Science and Metallurgy and Department of Earth Sciences, University of Cambridge, Cambridge, United Kingdom CB3 0FS

*Corresponding author information: Emilie Ringe <er407@cam.ac.uk>

S1. Influence of the amount of poly-2-vinylpyridine homopolymer on the self-assembly

Monomolecular films made of poly(styrene-b-2-vinylpyridine) (BCP), gold nanoparticles (AuNPs), and an increasing amount of h-P2VP were self-assembled to determine the amount of poly-2-vinylpyridine homopolymer (h-P2VP) needed to obtain sufficiently spaced NSs. Mean intermicellar distances were measured from transmission electron microscopy (TEM) images. The concentration of BCP remained the same (~1.8 mg/mL) and the amount of AuNPs was kept between 1.5 and 2.0 mg/mL. Constant surface pressure was applied between 10 mN/m and 15 mN/m during the transfer process.

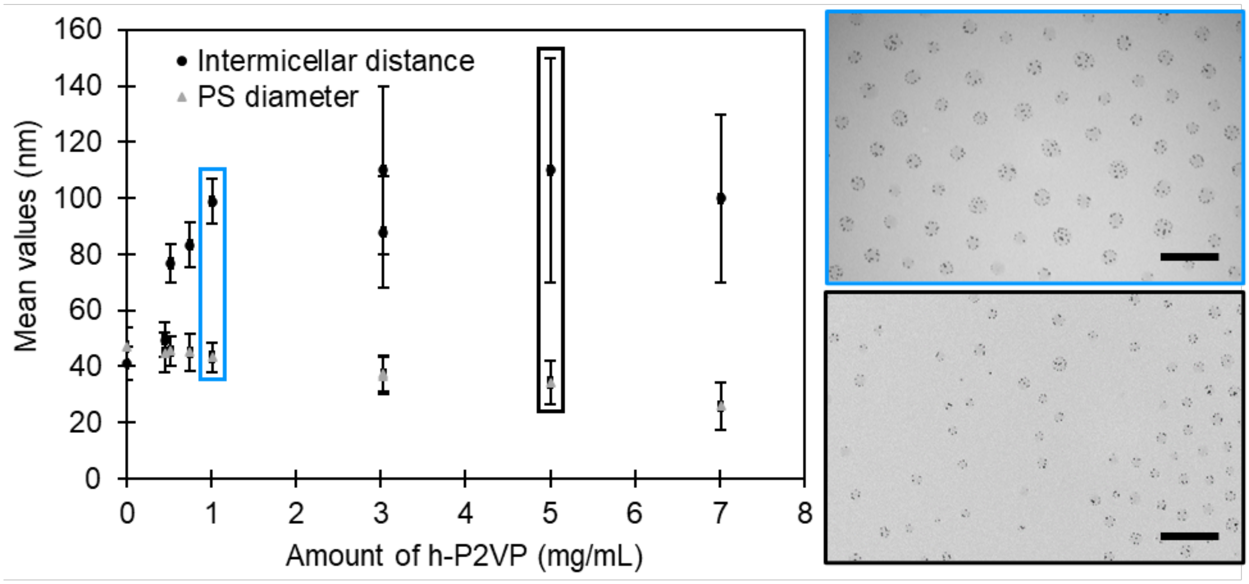

Figure S1. (a) Mean intermicellar distance and PS domain diameter as a function of the amount of h-P2VP added. Error bars represent the standard deviation determined from the measurement of 100 to 200 structures. (b) TEM images at two samples containing 1.0 mg/mL (blue) and 5.0 mg/mL of h-P2VP (black). Scale bars are 200 nm.

As shown in Figure S1, the mean intermicellar micelle distance increases with the amount of h-P2VP added until it reaches a maximum of around 100 nm. TEM images show that below 1 mg/mL, h-P2VP blends uniformly with the BCP, increasing the intermicellar distance without loss of the periodic structure. Above this value, regular micellar separation is lost, and monolayers exhibit large light gray regions without any AuNPs NSs, suggesting phase separation of h-P2VP from the P2VP blocks of the BCP. Some randomly isolated NSs can also be found with a spacing that overcome the diffraction limit of the optical microscope. Consequently, 3 mg/mL of h-P2VP is ideal to find sufficiently spaced CAs for the optical characterization. Since this addition of h-P2VP decreases the size of the polystyrene (PS) domains, a small amount of PS homopolymer (h-PS) is added to swell the hydrophobic domains so that the CAs are comparable to the close-packed ones. As demonstrated in the compression isotherm in Figure S2, the spread composite monolayers occupy a higher surface area at low surface pressure. A surface pressure of 1 mN/m is therefore chosen to increase the probability of finding sufficiently spaced CAs within a given sample.

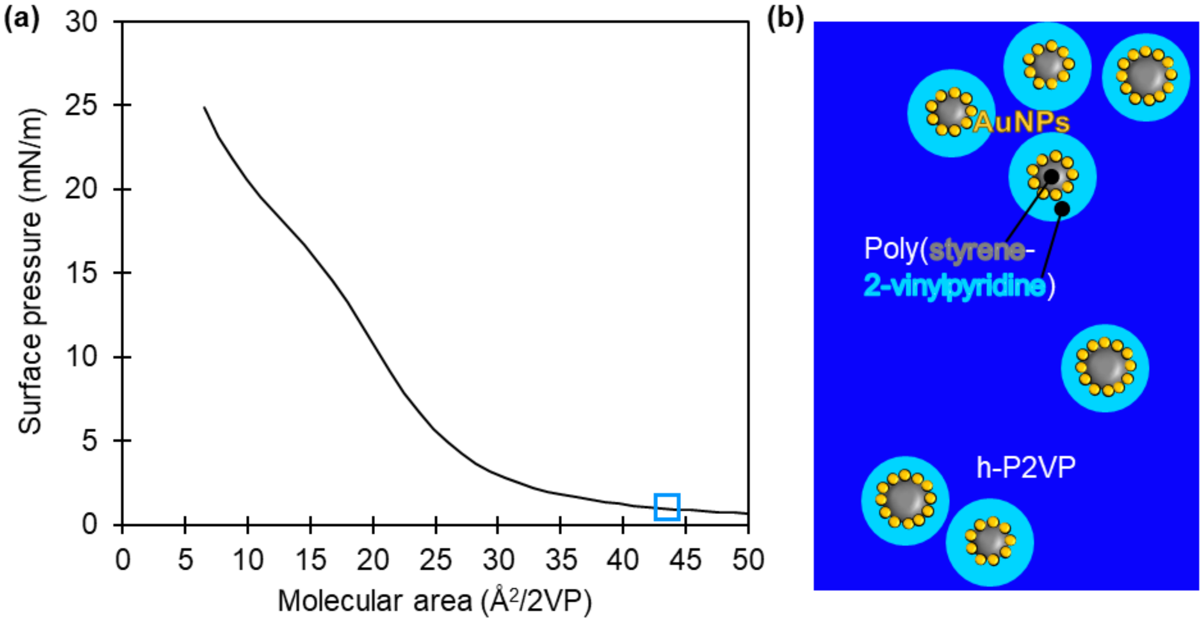

Figure S2. (a) Compression isotherm of a composite monolayer of BCP and h-P2VP, spread from chloroform solutions containing 1.81 mg/mL and 2.96 mg/mL of the two polymers, respectively, and (b) a schematic illustrating the self-assembled monolayer at the air-water interface with the presence of AuNPs at a surface pressure of 1 mN/m. The blue box in (a) represents the surface pressure employed to transfer the monolayer.

S2. Transfer of the self-assembled monolayers onto different surfaces

To investigate the substrate effect on self-assembled monolayers, thin layers of close-packed and distanced NSs were prepared with BCP alone and a blend of BCP with h-PS and h-P2VP. To form the densely packed NSs, 50 μL of a solution containing 1.8 mg/mL of BCP and 2.0 mg/mL of AuNPs solution was employed, and a surface pressure of 10 mN/m was used during the transfer. Another film with self-assembled NSs was formed with the same solution but transferred at 1 mN/m. The distanced NSs and the substrates were prepared as described in the Methods/Experimental section of the main paper. Monolayers were transferred on three different substrates: carbon-coated (C) glass, base-piranha-cleaned glass (G), and hybrid patterned glass/carbon (PG/PC). The number of NSs per unit area was measured from SEM images.

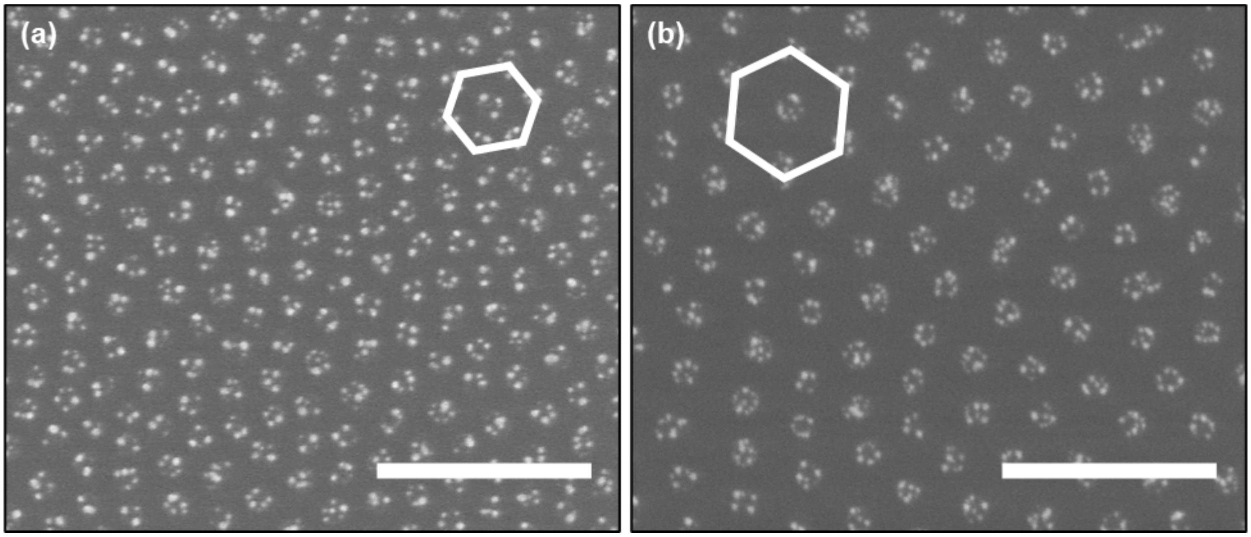

Figure S3. SEM images of Langmuir-Blodgett films composed of BCP and AuNPs transferred at 10 mN/m (a) and 1 mN/m (b) on a hybrid patterned glass/carbon substrate. Both images shown are on a carbon-coated section of the substrate with a scale bar of 500 nm.

CAs self-assemble at the air-water interface over a large range of surface pressures. The SEM images of CAs transferred at 10 mN/m and 1 mN/m, provided in Figure S3, show a hexagonal arrangement of NSs with a high degree of order, but which are more distanced from each other in the case of transfer at the lower surface pressure. Indeed, results presented in Figure S2 and Figure S3 confirm that the packing of CAs increases with the surface pressure. The reduction of the area occupied by the P2VP segments of the BCP upon compression can be explained by increased interdigitation at higher surface pressures. Furthermore, since the pKa of 2-vinylpyridine is 5.23[1] and the pH of the ultrapure water used in our laboratory is 5.83 ± 0.06, it is possible P2VP chains are partially protonated and thus slightly immersed in water through hydrophilic attractions. The immersion of the

P2VP segments can increase during monolayer compression, leading to more closely packed CAs.

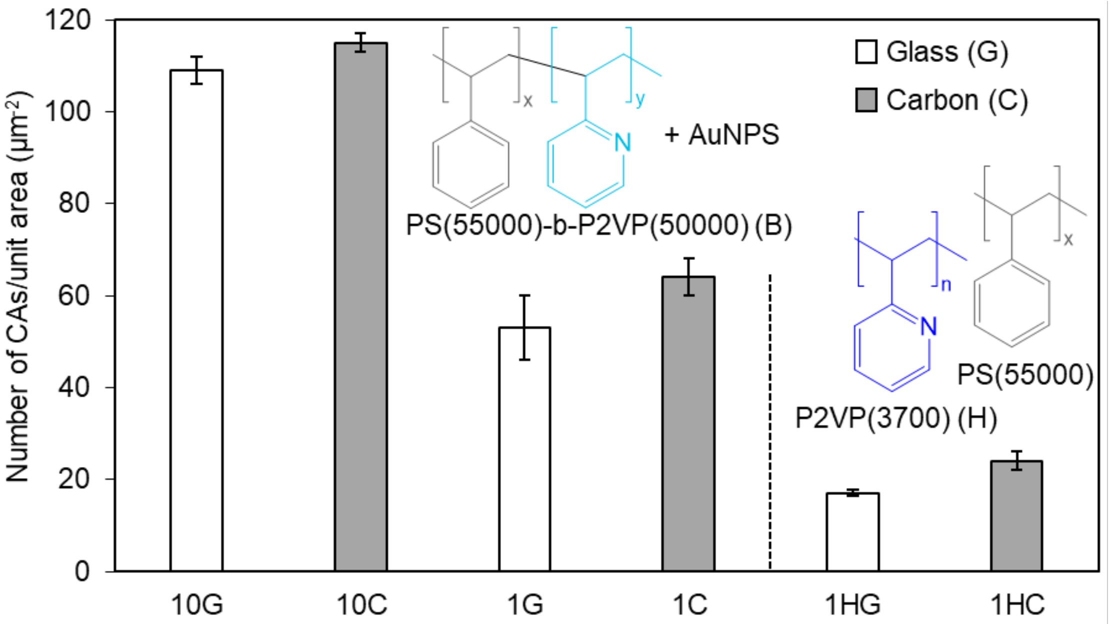

Figure S4. Histogram of the number of CAs per unit area self-assembled with BCP transferred at 10 mN/m (10) and 1 mN/m (1) on a hybrid patterned glass/carbon substrate measured on glass (G) and carbon (C), and a blend of BCP, h-PS and h-P2VP (H) deposited on a hybrid patterned glass/carbon at 1 mN/m (1) measured on glass (G) and carbon (C) respectively.

The histograms presented in Figure S4 also show how using h-P2VP in combination with a low surface pressure notably decreases the number of CAs per unit area. Interestingly, this value is slightly higher on carbon as compared to glass. It is possible that the adsorbed P2VP chains (from BCP and h-P2VP) are more extended on glass than on carbon through hydrogen bonding. Additionally, the dark field image shown in Figure S5 shows that the distribution of NSs is quite heterogeneous. Despite the higher density of NSs on carbon, the region appears darker since closer NSs have grown less than those separated on glass.

As a result, the scattering intensity of NSs on glass is higher since their dimensions are generally larger than those on carbon. Furthermore, the darkest areas on glass with fewer NSs suggests that the film may have torn during transfer or subsequent dewetting. In this case, glass region on the hybrid patterned carbon/glass substrate is the region of interest to find CAs with a spacing that allow for optical characterization beyond the diffraction limit.

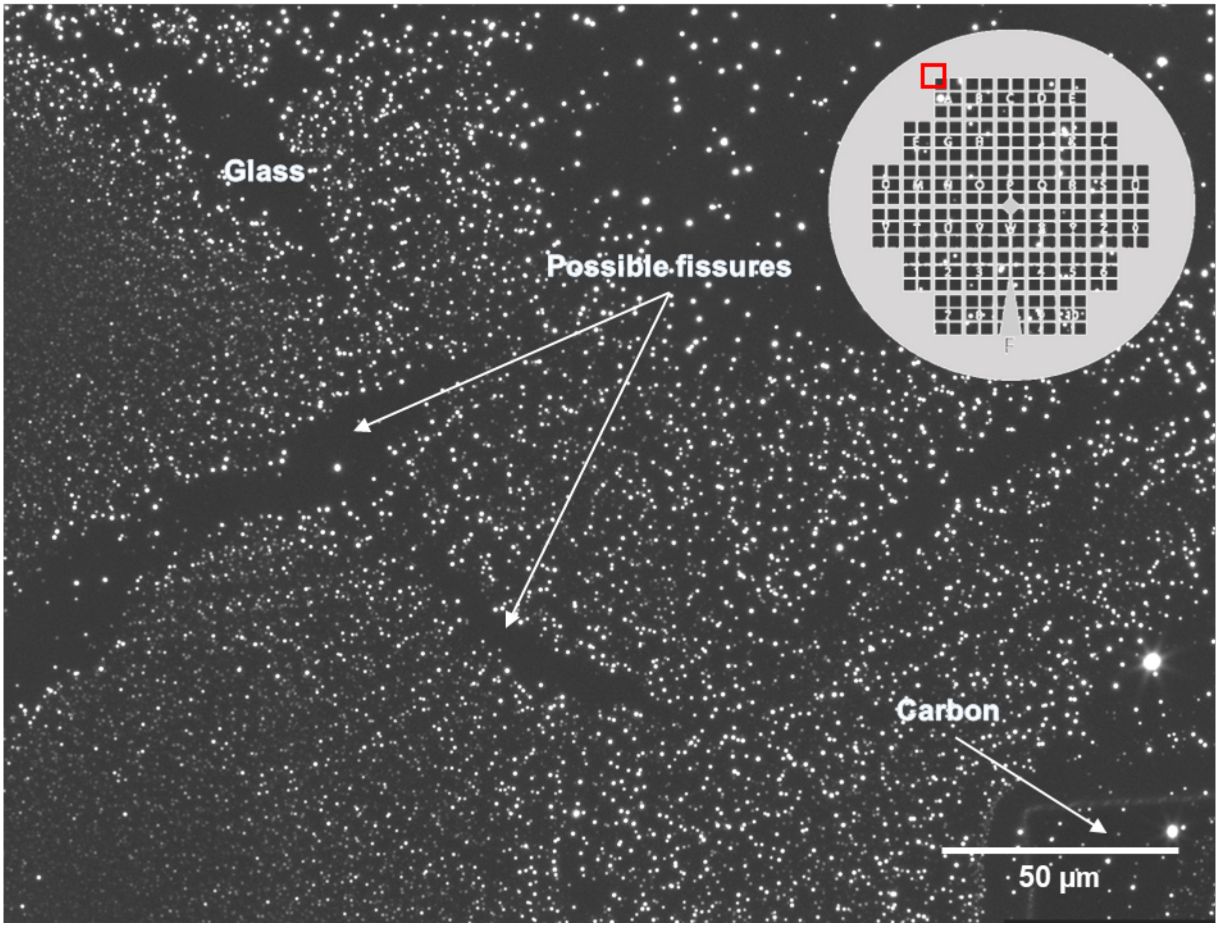

Figure S5. Dark field image of the self-assembled monolayer on a hybrid patterned glass/carbon substrate showing the scattering of the NSs along with the area analyzed on the grid motif. The main image presents NSs on glass and a little part of the carbon coating on the bottom right as pointed in the image.

S3. Scanning electron microscope images of the circular array of nanoparticles

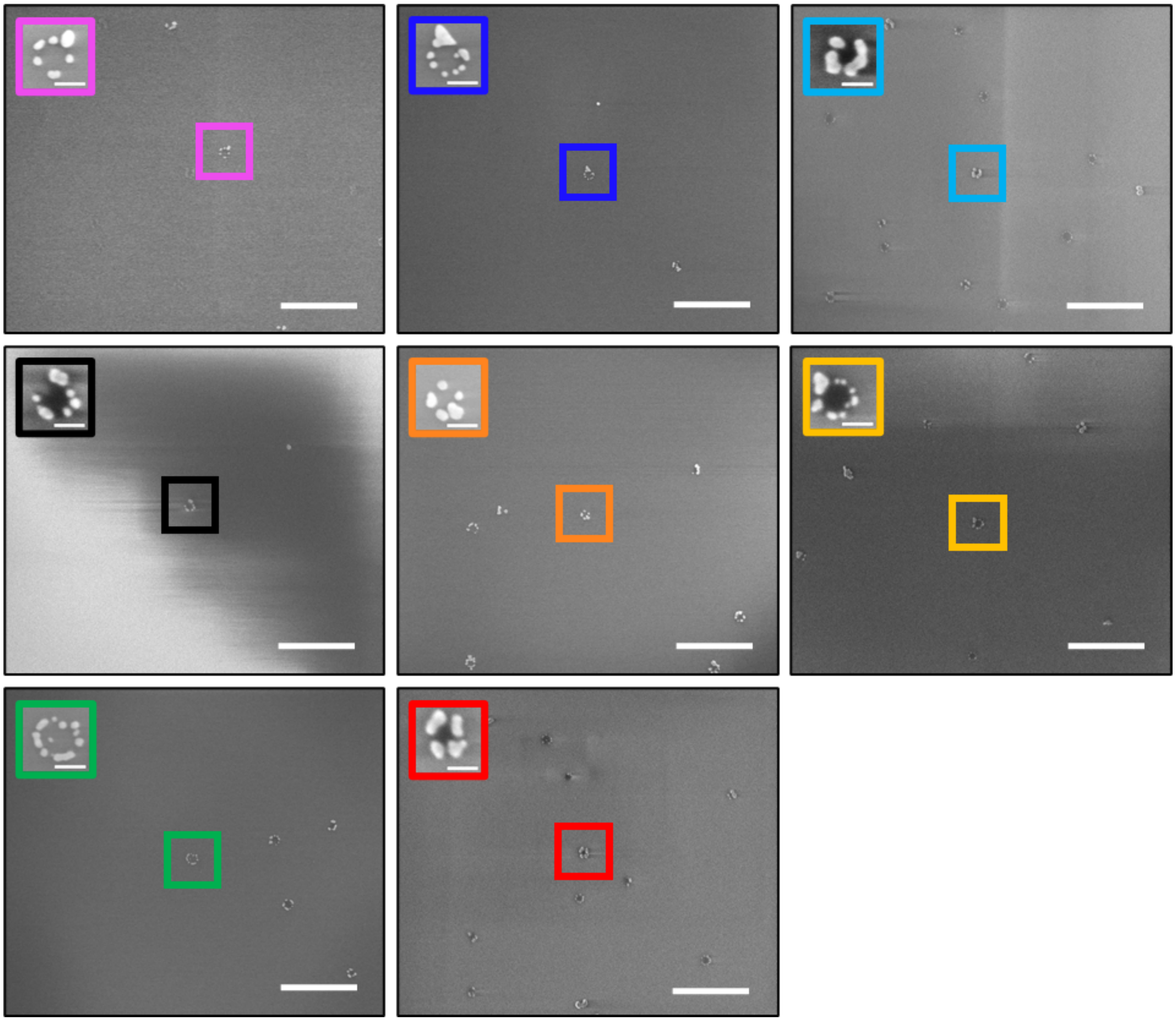

Figure S6. Large scale SEM images of individual circular array of gold nanoparticles (AuNPs) along with closer view color-coded images corresponding structures presented in the main paper. Inset scale bars are 100 nm and 1 um for the main images.

Scanning electron microscope images of the nanocrescents

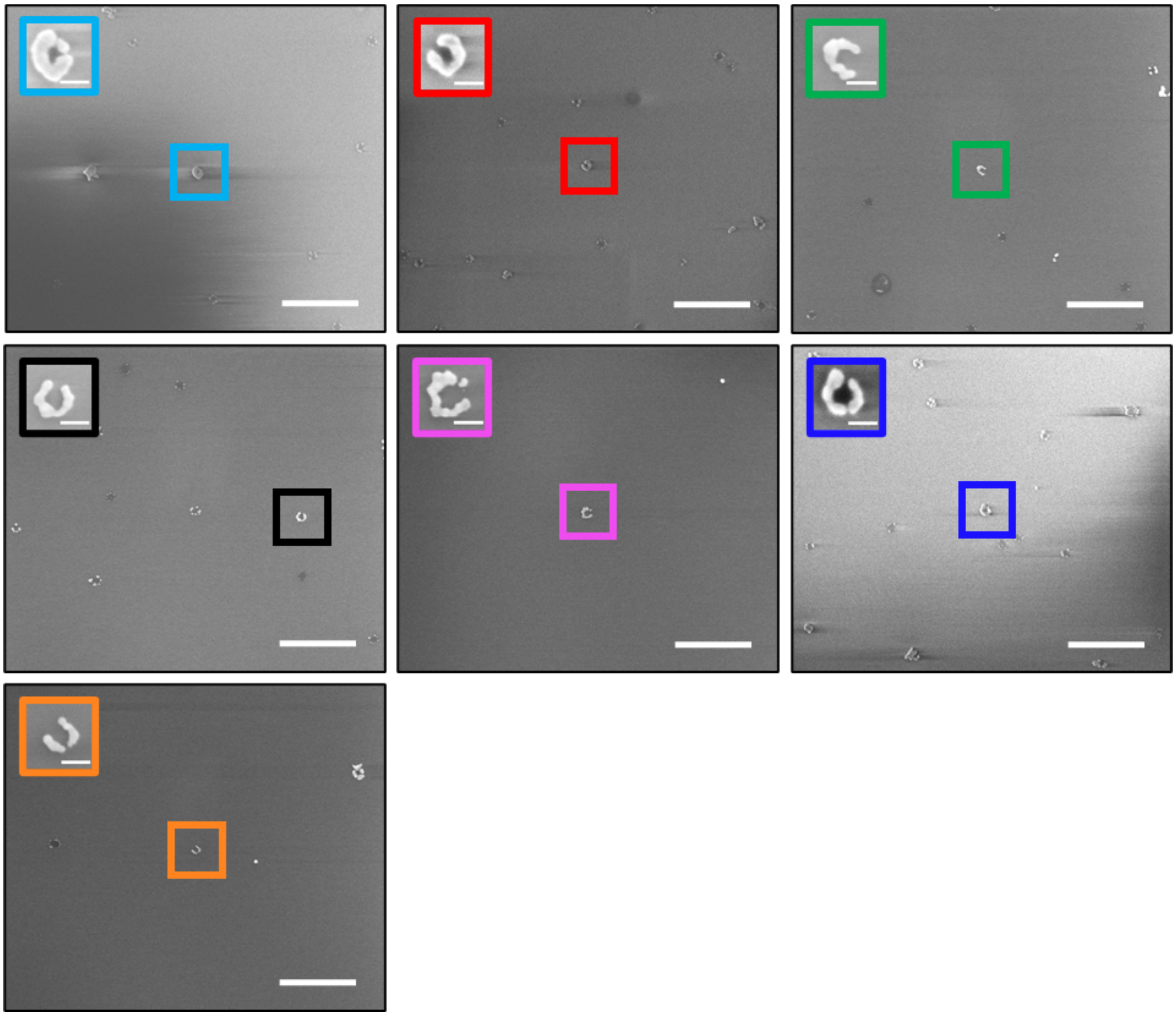

Figure S7. Large scale SEM images of individual gold nanocrescents along with closer view color-coded images of the corresponding structures presented in the main paper. Inset scale bars are 100 nm and 1 um for the main images.

Table S1: Dimensions of the nanocrescents and split-ring NSs, identified by their attributed colors.

Color-coded nanocrescents	Cavity diameter (nm)	Outer diameter (nm)	Width (nm)	Gap (nm)	Arc Length (nm)
Red	43	133	45	24	265
Light blue	41	157	58	12	304
Black	60	129	34	28	257
Pink	72	153	41	35	305
Green	56	124	34	51	231
Blue	63	145	41	17 and 7	176 and 112
Orange	56	117	30	47 and 11	116 and 87

Table S2: Arc length to width ratio and resonance positions of the nanocrescents and split-ring NSs, identified by their attributed colors.

Color-coded nanocrescents	Arc Length/Width	High Energy Position (nm)	Low Energy Position (nm)
Red	5.89	696	809
Light blue	5.26	720	767
Black	7.50	733	925
Pink	7.53	734	940
Green	6.79	-	878
Blue	4.29	711	915
Orange	3.83	745	-

S4 Nanostructure modeling

As mentioned in the Experimental section, structures were drawn using Autodesk AutoCAD 2020. First, the SEM image was imported into the CAD window. Then, a spline (SPLINE command) was used to trace the perimeter of NS components (isolated particles, fused particles, arcs, and crescents) and was subsequently extruded to create a 3D solid with a specific thickness t (EXTRUDE command). Corner rounding was then performed (FILLETEDGE command) where the fillet radius, r, was chosen to be half the thickness of

the structure, for example r = 5 nm was used for the paw structure with t = 10 nm. Note that it was impossible to set r = t/2 for the smallest particles in the necklace structure; the next smallest possible value was used. Finally, the CAD structure was exported as a lithography (.stl) file. A MATLAB script was used to import the lithography file, superimpose a cubic grid on the imported structure surface, identify the grid points within the surface boundaries and eventually create the dipole representation required by the electromagnetic simulation software DDSCAT.

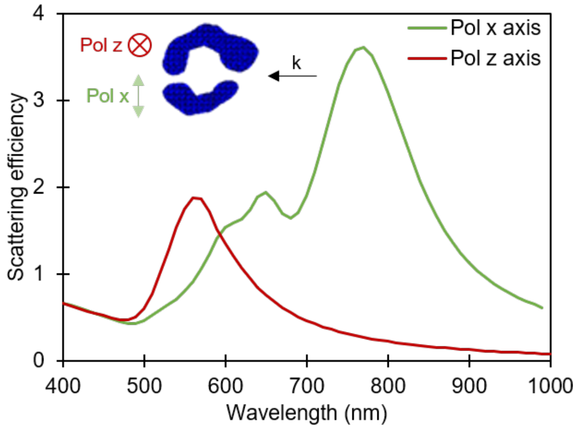

Figure S8. Calculated scattering spectra for in-plane (green) and out-of-plane (red) orthogonal polarizations for the illustrated split-ring structure. In both cases, the incident beam is in-plane as denoted by the wave vector k.

Table S3: Summary of the parameters used for the theoretical calculations for the necklace, paw, crescent, and split-ring structures. θE is the angle of the incident electric field from the surface plane.

<table>
<tr><th>Name of the structure</th><th>Thickness (nm)</th><th>Volume (nm^3)</th><th>Refractive index (RIU)</th><th>θE (°)</th><th>Dipole spacing (nm)</th></tr>
<tr><td rowspan="2">Necklace</td><td>10</td><td>50187</td><td rowspan="11">1.59</td><td rowspan="11">31</td><td rowspan="11">1-2</td></tr>
<tr><td>20</td><td>97521</td></tr>
<tr><td rowspan="3">Paw</td><td>10</td><td>48326</td></tr>
<tr><td>20</td><td>99395</td></tr>
<tr><td>30</td><td>157065</td></tr>
<tr><td rowspan="3">Crescent</td><td>20</td><td>165015</td></tr>
<tr><td>30</td><td>259277</td></tr>
<tr><td>40</td><td>323103</td></tr>
<tr><td rowspan="2">Split-ring</td><td>30</td><td>268531</td></tr>
<tr><td>40</td><td>471329</td></tr>
</table>

S5. Charge distributions calculated for normal incident radiation

Simulations presented in the main paper were carried for incident radiation with orthogonally polarized electric field directions oriented at an angle of 31° with respect to the substrate. This configuration was selected to approximate the light cone generated by the condenser of the darkfield microscope setup used to acquire the experimental spectra. Charge distributions for the 30-nm-height crescent and split-ring NS (Figure 9 of the main paper) were also calculated for normal incident light in order to obtain more symmetric maps and thus facilitate both the attribution of the individual modes and comparison with the literature. The results of these calculations are presented in Figures S9 and S10.

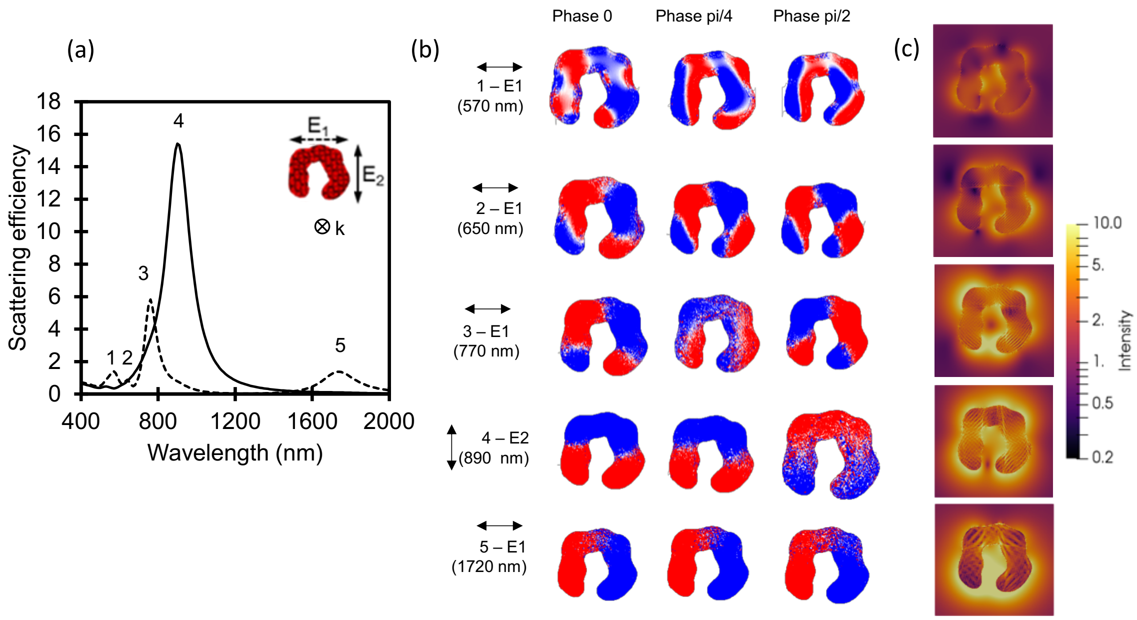

Figure S9. (a) Calculated scattering spectra for two orthogonal polarizations (solid and dashed lines) at normal incidence for the 30-nm-height crescent; (b) Charge distributions corresponding to the most prominent LSPR peaks visualized by plotting the dot product of the surface normal with the electromagnetic field vector at each point on the NP surface. Blue and red represent the opposite charges; (c) Calculated average field intensity over all phases (as opposed to field distributions for the phase with maximum intensity provided in the main paper).

Note that in the case of excitation perpendicular to the plane of the NS, the shoulder at ~750 nm (peak 3 in Figure 9a) is no longer observed indicating that its appearance is associated with the tilted incoming beam.

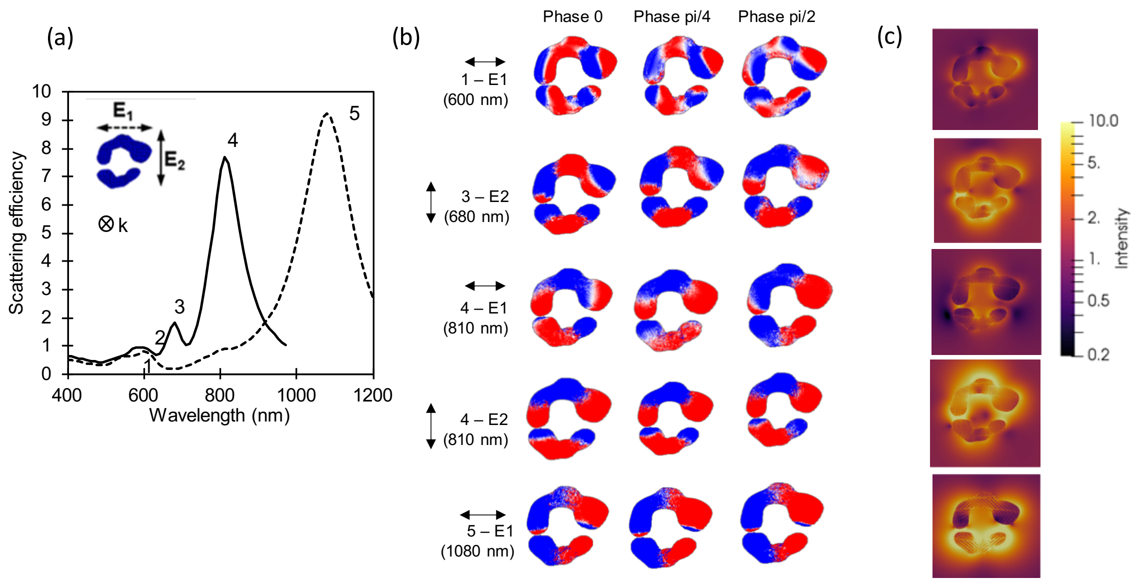

Figure S10. (a) Calculated scattering spectra for two orthogonal polarizations (solid and dashed lines) at normal incidence for the split-ring NS; (b) Charge distributions corresponding to the most prominent LSPR peaks visualized by plotting the dot product of the surface normal with the electromagnetic field vector at each point on the NP surface. Blue and red represent the opposite charges; (c) Calculated average field intensity over all phases (as opposed to field distributions for the phase with maximum intensity provided in the main paper). Note that in the case of excitation perpendicular to the plane of the NS, the resonance at 610 nm (peak 2 in Figure 9b) is no longer observed indicating that its appearance is associated with the tilted incoming beam.

S6. Field distributions of the paw and necklace structures for two electric field directions

The parameters used for the theoretical calculations are summarized in Table S2. In Figure S11, we present the calculated scattering spectra for the paw (orange) and necklace (green) structures. For the paw structure, the LSPR response arises from the coupling between the

dipolar plasmons from the neighboring nanoparticles (NPs) forming the ring. Additional coupling occurs between the two opposite NPs (particles I and III) contributing to the lowest energy peak (Figure S11a, number 5). In the necklace structure, various resonances also arise from the coupling of dipolar plasmons of nearest NPs. The coupling between smaller NPs gives rise to peaks at higher energy while elongated NPs generate peaks at lower energy. Peak 3-5 are triggered by the longitudinal mode of rod-like NPs. Since the NPs at the bottom of the NS is more distant from its adjacent NPs, the coupling is weaker, and so the corresponding LSPR energy is higher compared to the modes 4 and 5.

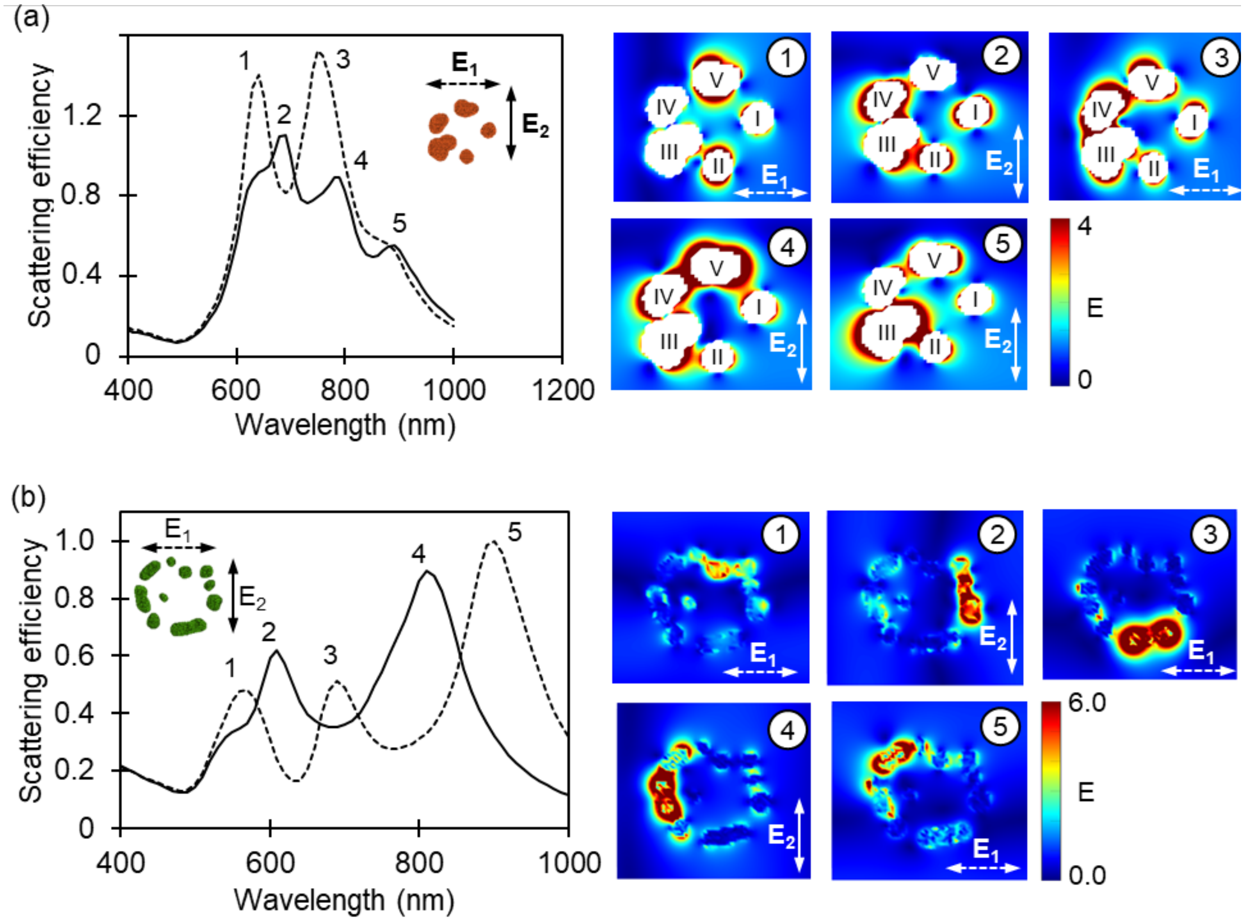

Figure S11. Calculated scattering spectra for two orthogonal polarizations (solid and dashed lines) for the paw (a) and necklace (b) structures along with field distributions corresponding to the most prominent LSPR peaks.

S7. Morphing from perfect to actual paw structure

To illustrate the importance of the irregular NP morphology, we calculated the change in the optical response as the paw structure gradually morphs from perfect disk-shaped NPs to the real structures. The software BLENDER was used to morph the shape from an ideal situation of perfect disk NPs to their actual shapes. The ideal shape was made by maintaining the geometric center of the particles, the same thickness, and the same volume for each particle.

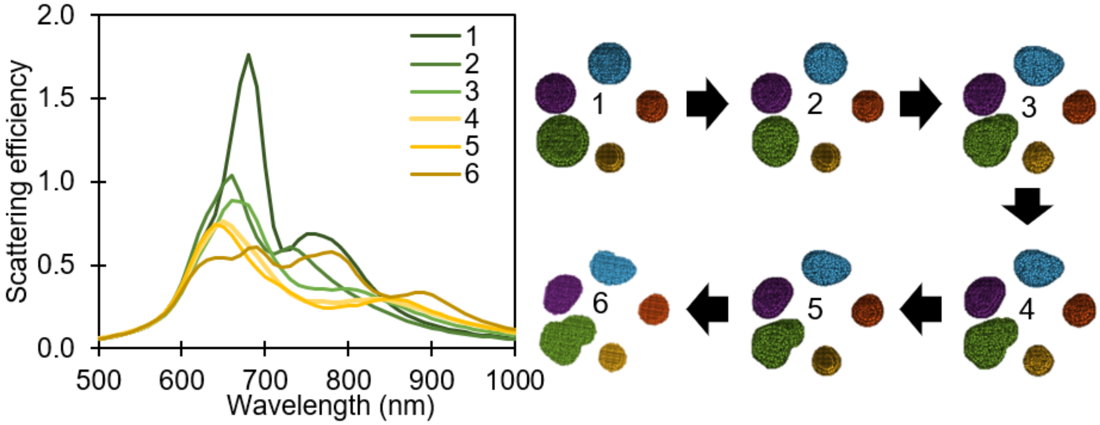

Figure S12. Morphological changes of the structure from regular disks (1) to the irregular particles (6) of the paw structure with their respective scattering spectra. The total volume, thickness and geometric centre of NPs are preserved.

As presented in Figure S12, the shape of the NPs affects the LSPR response of the whole structure presumably because of the changes in the interparticle distances. A smaller distance leads to enhanced NP interaction and a redshift of the low energy peaks, such as in the case of the red, blue, and purple NPs. Furthermore, the coupling between the green and red NPs becomes possible which explains the presence of a third peak at lower energy for the 6th structure. On the other hand, when the morphing decreases the interparticle distance, a blueshift of the higher energy peaks is observed. The concave angles formed on each elongated side of the green NP increase the interparticle distance with the adjacent purple and yellow NPs, and thus induce a weaker coupling.

REFERENCES

1. Kennemur, J. G. Poly(vinylpyridine) Segments in Block Copolymers: Synthesis, Self-Assembly, and Versatility. *Macromolecules* **2019**, *52*, 1354-1370.
